# Development and Validation of the UPLC-DAD Methodology for the Detection of Triterpenoids and Phytosterols in Fruit Samples of *Vaccinium macrocarpon* Aiton and *Vaccinium oxycoccos* L.

**DOI:** 10.3390/molecules27144403

**Published:** 2022-07-09

**Authors:** Rima Sedbare, Lina Raudone, Vaidotas Zvikas, Jonas Viskelis, Mindaugas Liaudanskas, Valdimaras Janulis

**Affiliations:** 1 Department of Pharmacognosy, Faculty of Pharmacy, Lithuanian University of Health Sciences, 50166 Kaunas, Lithuania; lina.raudone@lsmuni.lt (L.R.); mindaugas.liaudanskas@lsmuni.lt (M.L.); valdimaras.janulis@lsmuni.lt (V.J.); 2 Laboratory of Biopharmaceutical Research, Institute of Pharmaceutical Technologies, Lithuanian University of Health Sciences, 50166 Kaunas, Lithuania; vaidotas.zvikas@lsmuni.lt; 3 Institute of Horticulture, Lithuanian Research Centre for Agriculture and Forestry, 54333 Kaunas, Lithuania; jonas.viskelis@lammc.lt

**Keywords:** *Vaccinium macrocarpon*, *Vaccinium oxycoccos*, validation, UPLC-DAD, triterpenoids, phytosterol

## Abstract

Cranberries are used in the production of medicinal preparations and food supplements, which highlights the importance of triterpene compounds determination in cranberry fruit raw material. The aim of our study was to develop and validate for routine testing suitable UPLC-DAD methodology for the evaluation of triterpene acids, neutral triterpenoids, phytosterols, and squalene content in cranberry samples. The developed and optimized UPLC-DAD methodology was validated according to the guidelines of the International Council for Harmonization (ICH), evaluating the following parameters: range, specificity, linearity (R^2^ > 0.999), precision, LOD (0.27–1.86 µg/mL), LOQ (0.90–6.18 µg/mL), and recovery (80–110%). The developed and validated technique was used for the evaluation of triterpenic compounds in samples of *Vaccinium macrocarpon* and *Vaccinium oxycoccos* fruits, and their peels, pulp and seeds. The studied chromatogram profiles of *Vaccinium macrocarpon* and *Vaccinium oxycoccos* were identical but differed in the areas of the analytical peaks. Ursolic acid was the dominant compound in fruit samples of *Vaccinium macrocarpon* and *Vaccinium oxycoccos*. The highest amounts of triterpenic compounds were detected in the cranberry peels samples. The developed method for the detection of triterpene compounds can be applied in further studies for routine testing on the qualitative and quantitative composition of fruit samples of *Vaccinium macrocarpon* and *Vaccinium oxycoccos* species and cultivars.

## 1. Introduction

Small cranberry, or bog cranberry (*Vaccinium oxycoccos* L.), fruit raw materials are prepared in natural habitats, while American cranberry, or large cranberry (*Vaccinium macrocarpon* Aiton), is grown in private gardens and industrial plantations [1]. The plant raw material of cranberries is used in the production of cranberry juice, confectionery [2], food supplements [3], and medicinal products [4,5]. The biologically active compounds found in cranberry fruits (proanthocyanidins, anthocyanins, phenolic acids, flavonols, and triterpenoids [6]) have a variety of pharmacological effects, including antibacterial [5], antioxidant [7], anti-inflammatory [8], and anti-cancer [9] effects.

Triterpene compounds found in cranberry fruits are preserved in food products and dietary supplements made from cranberry fruits [10,11]. The qualitative and quantitative evaluation of triterpene compounds in crude fruits and their products may be one of the tools for determining quality in order to provide consumers with foods or preparations of high quality and of known composition. The highest levels of triterpene compounds in cranberry fruit samples are those of ursolic acid [12], which has been shown to have an anti-inflammatory effect [13] and to inhibit liver and breast cancer cell proliferation [14]. Two isomers of cis-3-*O*-*p*-hydroxycinnamoyl ursolic acid and trans-3-*O*-*p*-hydroxycinnamoyl ursolic acid have been found in cranberry fruits, which are less frequently detected in botanical raw materials [12,15]. Murphy et al. found that these identified ursolic acid esters inhibited the growth of tumor cells in the lung, cervix, breast, colon, and prostate cancers as well as in leukemia in vitro models [15]. He and Liu isolated β-Sitosterol-3-*O*-β-d-glucoside from cranberry fruit and found its inhibitory effect on the proliferative activity of human liver and breast cancer cells [14]. β-Sitosterol has been shown to have a similar structure to cholesterol and therefore acts as a cholesterol-reducing agent in competition with cholesterol in the human body [16]. β-Sitosterol has been shown to have antioxidant, antimicrobial [17], immunomodulatory, anti-inflammatory [18], and anticancer effects [19].

Modern instrumental methods of analysis and the latest methodologies are used for the qualitative and quantitative analysis of triterpene compounds in cranberries. They can be used to carry out routine research on cranberry plant raw materials and on the production and development of cranberry food supplements and medicines. Different methods of instrumental analysis for the analysis of triterpene compounds in cranberry plant matrix have been reported. According to literature data, the determination of the structure of triterpene compounds in cranberry fruit samples is performed by applying NMR spectroscopy [13,14,15]. The advantages of NMR spectroscopy are the determination of the molecular structure of triterpenoids, the simultaneous analysis of several compounds, and the non-destructive analysis of the molecules under study [19,20]. The application of NMR spectroscopy for the detection of triterpene compounds in complex matrices of plant raw materials can be complicated without prior sample cleaning and purification procedures, which, in turn, prolongs the time and cost of the analysis [19]. Klavins et al. applied gas chromatography with mass spectrometric detection (GC-MS) for the qualitative and quantitative evaluation of triterpene compounds in cranberry plant matrix [21,22]. The GC-MS method is used to detect complex compounds in plant matrices due to its selectivity and sensitivity, but derivatization of triterpene compounds in the sample is required to increase the volatility of the test compounds, which prolongs the assay time [20].

The HPLC method is used to investigate the qualitative and quantitative composition of pentacyclic triterpenoids in botanical raw materials and preparations. This method has advantages over GC in that it reduces the analysis time and achieves a higher resolution and sensitivity [20]. HPLC techniques with MS [6,12,13,15] and DAD [14,23] are used for the qualitative and quantitative evaluation of triterpene compounds in cranberry plant matrix. The application of the LC-MS methodology compared to the LC-DAD methodology has a higher sensitivity and yields more data on the structure of triterpene acids [24]. The determination of neutral triterpenoids and phytosterols by the LC-MS technique is complicated by the high lipophilicity of these compounds and their low content of ionizable functional groups [25,26]. Triterpenes do not have many chromophore groups in their molecular structure; thus, their detection using a DAD detector is performed at a non-specific wavelength of 200–210 nm [27,28], but this allows for the detection of lipophilic triterpenoids and sterols.

The aim of our study was to develop and validate a methodology for the detection of triterpene compounds in cranberry fruit samples that would be suitable for routine testing. Our proposed UPLC-DAD method allows for the detection of triterpene acids, neutral triterpenoids, phytosterols, and squalene in cranberry samples. Due to the lack of chromophores in the triterpene compounds, identification using a DAD detector was performed at a non-specific wavelength of 205 nm, and the UPLC-MS methodology was used for additional identification of the tirterpene compounds. The developed method for the detection of triterpene compounds can be applied in further studies on the qualitative and quantitative composition of fruit samples of *Vaccinium macrocarpon* and *Vaccinium oxycoccos* species and cultivars.

## 2. Results and Discussion

### 2.1. Optimization of Methods

The development of the UPLC-DAD methodology for the qualitative and quantitative analysis of triterpenoids and phytosterols in cranberry samples was performed, taking into account the composition of the mobile phase, flow rate, and temperature. The analytical separation was performed using an ACE C18 reversed-phase column (ACT, Aberdeen, UK; 100 × 2.1 mm, 1.7 μm particle size), which selectively separates polar and lipophilic non-volatile compounds [29,30].

**Figure 1:**
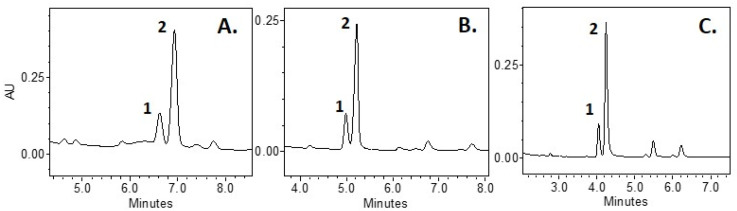
Chromatograms of 1-oleaonolic acid and 2-ursolic acid using different eluents, flow rate 0.2 mL/min. (**A**) Isocratic elution with 100% methanol (**A**) and 100% acetonitrile (**B**): 0 min, 15% A; 8 min, 15% A, 20 °C; (**B**) Isocratic elution with H_2_O (**A**) and 100% acetonitrile (**B**): 0 min, 12% A; 8 min, 12% A, 20 °C; (**C**) Gradient elution with 0.1% formic acid (*v*/*v*) (**A**) and 100% methanol (**B**): 0 min, 8% A; 8 min, 3% A, 25 °C.

The composition of the mobile phase and the choice of the type of elution are among the most important factors determining the efficient distribution of triterpene compounds [20]. In this study, we tested mobile phases of various compositions: acetonitrile/methanol, water/acetonitrile, and 0.1% formic acid/methanol. The lowest separation efficiency of oleanolic acid and ursolic acid from other analytes of cranberry fruit samples was found when using the acetonitrile/methanol mixture (Figure 1). Acidification of the aqueous medium with formic acid and the application of the gradient elution improved the resolution and the symmetry of the peaks (Figure 1C) [31]. Isocratic elution failed to isolate the more lipophilic compounds (neutral triterpenoids, phytosterols, and squalene), and thus other alternatives were sought to allow for the isolation of the compounds. The gradient elution with the 0.1% formic acid/methanol mixture increased the concentration of methanol in the solvent, thus increasing the lipophilicity of the solvent, allowing further isocratic elution with a mixture of 98% methanol and 2% of 0.1% formic acid to separate the peaks of neutral triterpenoids, phytosterols, and squalene. With a longer analysis time, the choice of methanol is better than that of acetonitrile, because this helps to avoid the irreversible absorption of matrix compounds in the column, which increases the stability of the column distribution [32].

Column temperature selection is an important tool in optimizing the suitability of the system and the quality of the distribution [33]. As the temperature increases, the viscosity of the solvents decreases, resulting in a change in the rate of analyte distribution in the column [34]. During the study, when selecting the system parameters of 20 °C to 35 °C and the flow rate of 0.1–0.4 mL/min, we found that the most efficient distribution of the analytes was achieved at 25 °C and 0.2 mL/min flow rate. During the study, the injection volume ranged from 1 µL to 3 µL, and for further analysis, an injection volume of 1 µL was chosen because higher injection volumes resulted in the blending of the adjacent peaks.

The best resolution for the determination of the analytes of triterpenoids and phytosterols in cranberry extracts was determined using a gradient composed of 0.1% formic acid (A) and 100% methanol (B) at a flow rate of 0.2 mL/min. The gradient change was the following: 0 min, 8% of A; 8 min, 3% of A; 9 min, 2% of A; 29.5 min, 2% of A; and 30 min, 8% of A. The next injection was delayed for 10 min to return to baseline concentrations of 8% of A and 92% of B. Applying this methodology, triterpene acid analytes were separated at 0 to 7 min, and from 10 to 24 min, analytes of neutral triterpenoids, phytosterols, and squalene were distributed.

### 2.2. Validation of Methods

#### 2.2.1. Specificity

Validation of the developed UPLC-DAD methodology was performed according to the guidelines of the International Conference on Harmonization (ICH) using the following analytical parameters: specificity, linearity, limits of detection and quantitation, precision, and accuracy [35].

**Figure 2:**
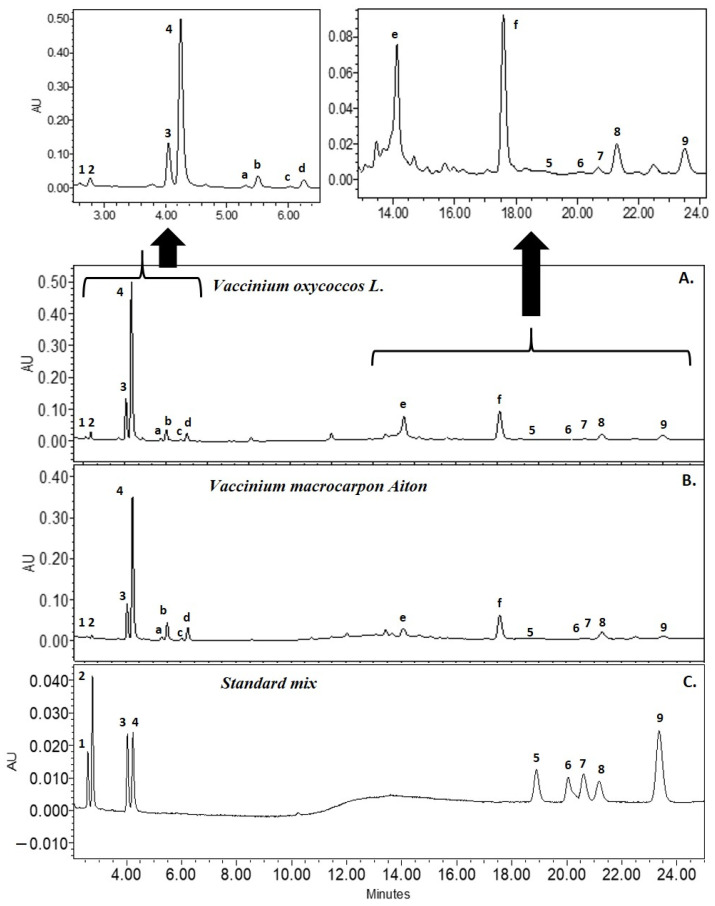
UPLC-DAD profile of cranberry fruit samples (**A**) and (**B**) and the mix of standards (**C**) at 205.5 nm. Chromatogram of the mix of standards (**C**): (1) maslinic acid 21 µg/mL, (2) corosolic acid 46 µg/mL, (3) oleanolic acid 27 µg/mL, (4) ursolic acid 25 µg/mL, (5) β-Amyrin 42 µg/mL, (6) campesterol 42 µg/mL, (7) α-Amyrin 42 µg/mL, (8) β-Sitosterol 42 µg/mL, and (9) squalene 21 µg/mL. Chromatogram of cranberry samples (**A**), (**B**): unidentified analytes a, b, c, d, e, f with λmax.

Identification and qualitative analysis of the compounds were performed by comparing the peak retention times, UV absorption, and MS spectra of the reference standards and the cranberry matrix. The obtained chromatographic analyte profiles of *Vaccinium ocyccocus* and *Vaccinium macrocarpon* were identical, except for the peak areas of the analytes (Figure 2A,B). The following compounds were identified using the developed methodology: (1) maslinic acid, (2) corosolic acid, (3) oleanolic acid, (4) ursolic acid, (5) β-amyrin, (6) campesterol, (7) α-amyrin, (8) β-Sitosterol, and (9) squalene (Figure 2). The rise of the baseline seen in the chromatogram (Figure 2) was due to a change in polarity and a higher methanol concentration, which resulted in a higher absorbance at non-specific UV wavelengths [32].

Not all of the detected analytes were identified in the chromatogram of cranberry sample extracts. Some of these analytes have characteristic UV absorption maxima (Figure 2a–f), which are determined by the presence of chromophoric groups in the structure of triterpene compounds [15,36]. Two ursolic acid esters have been described in the literature: cis-3-*O*-*p*-hydroxycinnamoyl ursolic acid and trans-3-*O*-*p*-hydroxylcinnamoyl ursolic acid, which have λmax 308.9 nm and λmax 312.4 nm, respectively [15]. The following compounds were found to have UV maxima in our study: (a) λmax 308.8, (b) λmax 308.8, (c) λmax 312.4 and (d) λmax 312.4. According to the literature, the major m/z fragment of cis-3-O-p-hydroxycinnamoyl ursolic acid and trans-3-*O*-*p*-hydroxylcinnamoyl ursolic acid was [M] − 601.4 [37]. In our study, after compound fragmentation, the relative ion content found by MS/MS detection was *m*/*z* 601.41 (100%), 144.98 (39.87%), 437.32 (4.32%), and 163.00 (1.43%). Fragments [M − H_2_O] − 144.98 and [M] − 163.00 could be identified as coumaric acid [38], and fragment [M − H_2_O] − 437.32 could be identified as ursolic acid or its isomer [39]. The λmax of compounds (e) and (f) was 281.4, but no data were found for compounds with identical UV spectra found in cranberries. The identification of specific triterpene compounds in cranberry samples could provide more insight into the specificity of the chromatographic profile of triterpene compounds in cranberries in the future.

#### 2.2.2. Linearity and LOD and LOQ

The linear range of the identified compounds, calibration Equations, their coefficients of determination, and limits of qualitative and quantitative determination are given in Table 1. The limits of linearity of the identified compounds (from 1563 to 2000 µg/mL) include all triterpenoid and phytosterol concentrations determined during the study. The coefficients of determination R^2^ were more than 0.999, which proves the linearity of the optimized methodology [40]. The calculated limits of detection (LOD) for the analytes ranged from 0.27 µg/mL to 1.86 µg/mL, and the limits of quantitation (LOQ) ranged from 0.90 µg/mL to 6.18 µg/mL.

#### 2.2.3. Accuracy

The accuracy parameter helps to evaluate the correspondence between the result obtained and the true value by expressing the closeness between the obtained results in percentage [41]. The recovery of the UPLC-DAD methodology was assessed by determining the amount of reference standards at three concentration levels: level 1 (95.47–107.96%), level 2 (100.52–106.12%), and level 3 (99.87–104.95%) with the relative standard deviation ranging from 0.03% to 5.83% (Table 2). The accuracy of the analytes obtained did not exceed the limit of 80–110% specified in the European Commission Directive 96/23/EC [42].

#### 2.2.4. Precision

The precision of the methodology was assessed based on repeatability and intermediate precision in determining the variation of the identified compounds in *Vaccinium oxycoccos* and *Vaccinium macrocarpon* fruit samples (Table 3). In the fruit samples of *Vaccinium oxycoccos* and *Vaccinium macrocarpon*, the relative standard deviations of retention time in intra-day precision ranged from 0.06% to 0.11% and from 0.20% to 0.88%, and in the inter-day precision, from 0.25% to 0.60% and from 0.31% to 0.68%, respectively. Concerning the %RSD of the content of the identified triterpenoids, phytosterols, and squalene in fruit samples of *Vaccinium oxycoccos* and *Vaccinium macrocarpon*, the intra-day precision ranged from 0.78% to 3.16% and from 0.90% to 8.81%, and the inter-day precision ranged from 1.71% to 8.88% and from 1.59% to 11.20%, respectively. The HortRat values of inter-day precision found in *Vaccinium oxycoccos* and *Vaccinium macrocarpon* fruit samples ranged from 0.30 to 1.05 and from 0.30 to 1.01, respectively. The HorRat values found in cranberry fruit samples fell within the recommended range (0.3–1.3) of HorRat values set in the single-laboratory validation studies [43]. The results of the suitability of the UPLC-DAD methodology showed that the selected chromatographic parameters were suitable for the qualitative and quantitative evaluation of the triterpene compounds and phytosterols of the analyzed cranberry plant extracts.

### 2.3. Investigation of the Qualitative and Quantitative Composition of Triterpene Compounds and Phytosterols in Different Parts of Cranberry Fruit

The developed and validated UPLC-DAD methodology was applied to the qualitative and quantitative analysis of triterpenoids and phytosterols in *Vaccinium macrocarpon* and *Vaccinium oxycoccos* cranberry fruits and their parts. The test samples of large cranberries (*Vaccinium macrocarpon*) consisted of the whole cranberry berry, the peel, the juicy pulp of the berry, and the seeds. In addition, three test samples of small cranberries (*Vaccinium oxycoccos*) were prepared: the whole cranberry berry, the peel, and the juicy berry pulp with seeds. The juicy pulp of *Vaccinium oxycoccos* cranberry fruit contains a higher amount of water (84.2–98.8%) than the pulp of *Vaccinium macrocarpon* fruit (84.8–88.0%) does [44]; therefore, it is difficult to separate the pulp from the seeds without losing the pulp mass. As a result, only the peels were mechanically separated.

The outer surface of the berry peels is covered with a waxy layer, which protects the fruit from water evaporation, temperature changes, UV rays, and microorganisms [22]. About 50% of the waxy layer of cranberry berries consists of triterpenoids, which determine the various biological effects of preparations made from cranberry fruits [45,46]. Qualitative and quantitative analysis of triterpenes are relevant in assessing the quality of cranberry raw material and cranberry preparations. In our study, peel samples of the fruit of *Vaccinium macrocarpon* and *Vaccinium oxycoccos* were found to contain the highest total amount of the identified triterpene compounds and phytosterols (accordingly, 11,109.86 ± 166.27 µg/g and 9582.45 ± 143.74 µg/g) (Table 4). The content of triterpene compounds and phytosterols in cranberry fruit samples was by about 1.2 times lower than that found in peel samples. The slight difference in the levels of triterpene compounds and phytosterols between the peel samples and the whole cranberry fruit samples was due to the fact that most of the mass of the raw material was composed of fruit peels, as water was removed from the berry samples during the lyophilization process [44]. Samples of the large cranberry (*Vaccinium macrocarpon*) cultivar ‘Stevens’ had higher total levels of triterpenoids and phytosterols than samples of the small cranberry (*Vaccinium oxycoccos*) fruit did (Table 4). During the validation of the method for the detection of triterpene compounds in the precision test (Table 3), the total content of triterpene compounds and phytosterols (11,779.28 ± 196.13 µg/g) found in fruit samples of small cranberry was higher than that (7973.65 ± 134.46 µg/g) found in fruit samples of the large cranberry ‘Red Star’ cultivar. The variability of triterpene compounds in fruit samples of large cranberries and small cranberries may have been due to geographical, climatic, and genetic factors [22,47].

The ursolic acid content in fruit samples of large cranberries and small cranberries was, respectively, 62.64% and 64.80% of the amount of the identified triterpenoids and phytosterols. The maximum levels of ursolic acid found in the fruit peel samples of small cranberries and large cranberries were 5809.40 ± 87.14 µg/g and 6612.79 ± 99.19 µg/g, respectively (Table 4). Klavins et al. found a higher amount (66,000 µg/g) of ursolic acid in the waxy layer extract of large cranberry fruits [22]. In a study by Xue et al., the ursolic acid content in fruit samples of large cranberries ranged from 28,920 µ/g to 109,180 µ/g [48]. Lower ursolic acid levels were found in fresh fruit samples of large cranberries and small cranberries (respectively, 930–1090 µg and 120 µg/g) [12].

The amount of oleanolic acid in fruit samples of large cranberries and small cranberries was, respectively, 17.42% and 18.82% of the identified triterpenoids and phytosterols. The largest amount of oleanolic acid found in samples of large cranberry fruit peels was 2562.29 ± 38.43 µg/g. A similar amount of oleanolic acid (2046.18 ± 30.69 µg/g) was found in samples of small cranberry fruit peels. The amount of oleanolic acid in cranberry fruit samples detected by Oszmianski et al. (894–1137 µg/g) was similar to that found in our study [47]. Meanwhile, Xue et al. found larger amounts of oleanolic acid in cranberry fruit samples (5910–31,240 µg/g) [48].

The largest amounts of maslinic acid found in peel samples of large cranberries and small cranberries were, accordingly, 112.45 ± 2.69 µg/g and 106.73 ± 1.60 µg/g. No maslinic acid was detected in cranberry pulp and seed samples. The maximum levels of corosolic acid found in fruit peel samples of large cranberry and small cranberry were, respectively, 241.38 ± 3.69 µg/g and 281.62 ± 4.22 µg/g. No corosolic acid was detected in cranberry pulp and seed samples. In a study by Xue et al., the content of maslinic acid in cranberry fruit extract varied from 320 µg/g to 900 µg/g, and the content of corosolic acid ranged from 1190 µg/g to 3810 µg/g [48].

The largest amounts of α-Amyrin found in fruit peel samples of large cranberries and small cranberries, were, respectively, 249.53 ± 3.74 µg/g and 106.91 ± 1.60 µg/g. No α-Amyrin was detected in cranberry pulp and seed samples. The highest β-Amyrin content found in large cranberry seeds was 336.74 ± 5.05 µg/g. No β-Amyrin was detected in the *Vaccinium oxycoccos* samples. Klavins et al. found the cuticle wax extract of large cranberry fruits to contain 2500 µg/g of α-Amyrin and 3000 µ/g of β-Amyrin [22]. In another study, Klavins et al. found that the amount of α-Amyrin in small cranberry fruit extract was 7800 µg/g, and the amount of β-Amyrin was below detection limits [46]. In large cranberry samples, the authors detected 9200 µg/g of β-Amyrin but did not detect any α-Amyrin [46].

The β-Sitosterol content in the samples of large cranberries and small cranberries was, respectively, 14.65% and 13.25% of the total content of triterpene compounds and phytosterols. No statistically significant differences were found in the amount of β-Sitosterol between *Vaccinium oxycoccos* peel and seed pulp samples (Table 4). The content of β-Sitosterol in the peel and pulp samples of *Vaccinium macrocarpon* did not differ statistically significantly, while the content of β-Sitosterol in the seed samples was about 1.6 times higher. The levels of β-Sitosterol in different parts of the fruit did not differ significantly because β-Sitosterol is part of the lipid bilayer of the plant cell membrane involved in regulatory and structural cell functions [49]. Campesterol was detected only in large cranberry seed samples (66.55 ± 1.56 µg/g), while in samples of other cranberry parts, the content of campesterol was below the LOQ value. In a study by Klavins et al., the β-Sitosterol content of 3150 µg/g found in the extract of the waxy layer of large cranberry fruit was higher than the amount of β-Sitosterol found in our study [22]. Meanwhile, in a study by Nilova et al., the level of β-Sitosterol (4 µg/g) found in berry press residues of cranberries was significantly lower than that detected in our study [50].

The highest amount of squalene found in large cranberry seed samples was 1116.90 ± 16.75 µg/g. Squalene is a precursor to triterpenoids and phytosterols and in plants is commonly found in seeds and oils [51]. Squalene has cholesterol-lowering, antioxidant, anticancer, and antibacterial effects [52].

The ursolic and oleanolic acids found in cranberry fruit samples have antioxidant [6], anti-inflammatory [13], and anti-cancer [15] activity, thus their presence in foods or food supplements is important for disease control, prevention, and prophylaxis. The highest levels of ursolic acid and other triterpenoids were found in the waxy layer of cranberry peels, thus the content of ursolic acid and other triterpenoids in cranberry products depends on the method of fruit processing [12]. The description of the composition of cranberry preparations often does not indicate from which raw material (whole fruit, juice, or extract) of the cranberry plant it is made or what the qualitative and quantitative composition of the biologically active compounds is [11,53]. The fruits of *Vaccinium oxycoccos* harvested in natural habitats and the fruits of *Vaccinium macrocarpon* harvested from cranberry plantations are morphologically similar and have similar chromatographic profiles of biologically active compounds, which, however, differ in quantitative composition. The development and refinement of new and innovative methodologies is important for supplying customers with cranberry products and preparations of known composition and a high quality. Qualitative and quantitative determination of triterpene compounds in cranberry raw material and products may be an important tool in determining the authenticity of cranberry raw material and the composition of cranberry products in the development of value-added health promotion products.

## 3. Materials and Methods

### 3.1. Reagents

Acetone (manufacturer: Sigma-Aldrich, Steinheim, Germany), methanol (manufacturer: Sigma-Aldrich, Steinheim, Germany), formic acid (manufacturer: Merck, Darmstadt, Germany), acetonitrile (manufacturer: Sigma-Aldrich, Steinheim, Germany), reference standards for maslinic acid, corosolic acid, oleanolic acid, ursolic acid, β-Amyrin, campesterol, α-Amyrin, and β-Sitosterol were obtained from Sigma-Aldrich (Steinheim, Germany), and reference standards for squalene were obtained from Carl Roth (Karlsruhe, Germany). The purified deionized water used in the experiments was prepared using a Milli-Q^®^ (Millipore, Bedford, MA, USA) water purification system.

### 3.2. Raw Material

The object of the study was samples of mature and ripe fruit of *Vaccinium macrocarpon* and *Vaccinium oxycoccos*. Samples of the *Vaccinium macrocarpon* cultivar ‘Red Star’ and *Vaccinium oxycoccos* cranberry fruit used for the development and validation of the methodology were obtained from, respectively, the Botanical Garden of Vilnius University, Kairėnai, Vilnius (54°43′47.1″ N 25°24′34.9″ E) and Dubravai, Kaunas distr. (54°50’54.5” N 24°04′35.2″ E). Parts of cranberries were obtained by mechanical separation of peel, pulp, and seeds of *Vaccinium macrocarpon* cultivar of ‘Stevens’ collected in Giedručiai, Šakiai district. (54°57′50.8″ N 23°01′34.7″E) and samples of *Vaccinium oxycoccos* collected in Čepkeliai (53°59′07.3″ N 24°26′48.6″ E). The collection time was September 2021. Cranberry fruits were ground and frozen at −60 °C in an ultra-low-temperature freezer (CVF330/86, ClimasLab SL, Barcelona, Spain). Cranberry fruits were freeze-dried according to the methodology described by Gudžinskaitė et al. [54]. The fruits were powdered in a Retsch GM 200 electric mill (Retsh GmbH, Hahn, Germany). Loss on drying was determined using the method described in the European Pharmacopoeia Ph.Eur.01/2008: 20232 [55].

### 3.3. Preparation of Cranberry Extracts

About 1 g (precise weight) of the lyophilized cranberry powder was extracted with 10 mL of acetone in an Elmasonic P ultrasonic bath (Singen, Germany) at 80 kHz and 1130 W at 22 ± 1 °C to 60 ± 1 °C temperature for 60 min. After the extraction, the acetonic extracts were filtered into a 10-mL volumetric flask. The prepared extracts were stored in dark glass vials at 5–8 °C. Prior to the chromatographic analysis, the acetonic extracts were filtered through membrane filters with 0.22 µm pore size (Carl Roth GmbH, Karlsruhe, Germany).

### 3.4. Chromatographic Analysis

The analysis of the qualitative and quantitative composition of triterpenic compounds, sterols, and squalene in cranberry fruit was performed using a Waters ACQUITY Ultra High-Performance LC system (Water, Milford, MA, USA) with a photodiode array detector and Xevo TQD tandem mass spectrometer (Water, Milford, MA, USA). An Avantor ACE Excel UHPLC column (C18, 100 × 2.1 mm, 1.7 µm particle size) was used for the separation of the compounds at 25 °C. The injection volume was 1 µL, and the distribution was performed using 0.1% formic acid (*v*/*v*) (A) and 100% methanol (B) at a flow rate of 0.2 mL/min and the following gradient change: 0 min, 8% A; 8 min, 3% A; 9 min, 2% A; 29.5 min, 2% A; and 30 min, 8% A, delaying the subsequent injection by 10 min. Mass spectrometer conditions were used as follows: a negative electrospray ionization mode with 3 kV capillary voltage and 50 V cone voltage, the source temperature was set to 150° C, the desolvation gas temperature to 600° C, flow to 1000 L/h, and cone gas flow to 20 L/h. MS analysis was performed in negative scan mode in *m*/*z* range from 110 to 1000. MS/MS analysis was performed in product ion scan at 40 eV collision energy.

### 3.5. Development and Validation of the Method

The UPLC-PDA method was developed considering the composition of the mobile phase, the elution gradient, the duration of the analysis, the flow rate, the injection volume, the column parameters, and the optimal temperature. For validation, a methodology was selected in which the triterpenoids, sterols, and squalene analytes in *Vaccinium macrocarpon* and *Vaccinium oxycoccos* fruit matrixes were best separated from each other in the chromatogram.

The validation of the method was performed according to the guidelines of the International Council for Harmonization (ICH), the acceptance criteria of the validation were selected considering the recommendations of Eurochem, the Guidelines for Standard Method Performance Requirements, and the Guidelines for Dietary Supplements and Botanicals [35,43,56,57]. The following parameters were evaluated during the validation: accuracy, precision (repeatability and intermediate precision), specificity, limit of detection (LOD), limit of quantification (LOQ), linearity, and range of determination.

Specificity was determined by comparing the analyte retention time and the MS/MS spectrum between the reference standard and the cranberry matrix. Linearity was determined by constructing calibration regression equations consisting of 6 to 10 points and measuring standard solutions of known concentration. To evaluate linearity, the coefficient of determination (R^2^) was set (suitable when R^2^ > 0.999). The limit of detection (LOD) and the limit of quantitation (LOQ) were determined at a signal-to-noise (S/N) ratio of 3:1 and 10:1, respectively. Precision was determined by evaluating the relative standard deviation (RSD%) of the retention time and the amount of 6 *Vaccinium macrocarpon* extracts and 6 *Vaccinium oxycoccos* extracts (Formula 1). The RSD% of repeatability was calculated from measurements taken on the same day (*n* = 6), and the intermediate precision was determined by analyzing six cranberry extracts for three consecutive days (*n* = 18). The acceptable precision value was determined by calculating Horwitz Ratio (HorRat) (Formula 2) [43].

RSD% = SD/m × 100 (1)

HortRat = RSD%/RSDr% = SD/m × 100/2C^(−0.15)(2)

(RSD%—relative standard deviation, SD—standard deviation, m—mean, RSDr%—acceptable precision value, and C—concentration expressed in parts of mass (g/g)).

Recovery was determined by adding a known amount of reference standards to a blank matrix at three levels: level 1—low concentration, level 2—medium concentration, and level 3—high concentration. Concentrations of level 1 were 10 µg/mL for all standards. Concentrations of level 2 were 250 µg/mL for oleanolic acid, 800 µg/mL for ursolic acid and 50 µg/mL for maslinic acid, corosolic acid, β-amyrin, campesterol, α-amyrin, β-sitosterol, and squalene. Concentrations of level 3 were 500 µg/mL for oleanolic acid, 1600 µg/mL for ursolic acid, and 150 µg/mL for maslinic acid, corosolic acid, β-amyrin, campesterol, α-amyrin, β-sitosterol, and squalene. Recovery was calculated according to Formula (3) (where Xp is the predicted concentration, and X1 is the measured concentration). Recovery was regarded as adequate when the Recovery% was in the range of 80–110% [42].

Recovery% = Xp/X1 × 100 (3)

### 3.6. Statistical Analysis

Data analysis was performed using computer software programs Microsoft Excel 2016 (Microsoft, Redmond, WA, USA) and SPSS Statistics 21 (IBM, Armonk, NY, USA). During the study, means, standard deviations (SD), and relative standard deviations (RSD) of the three independent evaluations were calculated. Linear regression analysis was performed to calculate the coefficient of determination R^2^ and to construct calibration equations for the calculation of the amounts of the identified compounds. To evaluate the difference in the amounts of triterpenic compounds between samples of cranberry, one-way analysis of variance ANOVA with Tukey’s test for multiple comparisons (the significance level set at 0.05) was used.

## 4. Conclusions

A UPLC-DAD method was developed and validated to detect triterpene acids, neutral triterpenoids, phytosterols, and squalene in a single assay. The validation parameters of the methodology—specificity, linearity (R^2^ > 0.999), precision, LOD (0.27–1.86 µg/mL), LOQ (0.90–6.18 µg/mL), and accuracy (95.47–107.91%)—met the requirements of the normative documents and confirm the suitability of the methodology for application.

The studied chromatogram profiles of *Vaccinium macrocarpon* and *Vaccinium oxycoccos* were identical but differed in the areas of the analytical peaks. The determination of triterpene compounds and phytosterols in different parts of cranberry fruit provides knowledge about the distribution of these compounds in cranberry organs and allows for a better understanding and prediction of how technological processes of cranberry fruit preparation and processing determine the content of triterpene compounds and phytosterols in cranberry foods and food supplements.

## Figures and Tables

**Table 1:** Parameters of the limits of linearity, detection, and quantitation of the identified compounds.

No.	Compound	Linear Range (µg/mL)	Calibration Equation	R^2^	LOD (µg/mL)	LOQ (µg/mL)
1	Maslinic acid	3.1–200	y = 2790x + 3990	0.9994	0.89	2.95
2	Corosolic acid	3.1–200	y = 3280x + 750	0.9997	0.66	2.21
3	Oleanolic acid	2.3–600	y = 3240x +1.29 × 10^4^	0.9997	0.55	1.85
4	Ursolic acid	3.9–2000	y = 2930x + 3.9 × 10^4^	0.9999	0.54	1.81
5	β-Amyrin	6.3–200	y = 3170x + 6470	0.9998	1.11	3.69
6	Campesterol	6.3–200	y = 2910x + 1350	0.9995	1.80	5.99
7	α-Amyrin	6.3–200	y = 3090x − 1030	0.9996	1.37	4.58
8	β-Sitosterol	6.3–200	y = 2100x + 4830	0.9995	1.86	6.18
9	Squalene	1.6–200	y = 3.09 × 10^4^x + 5.04 × 10^4^	0.9999	0.27	0.90

**Table 2:** Determination of the recovery of the identified compounds.

No.	Compound	1 Level (Low Concentration of Range) ^1^		2 Level (Medium Concentration of Range) ^2^		3 Level (High Concentration of Range) ^3^	
		% Recovery	% RSD	% Recovery	% RSD	% Recovery	% RSD
1	Maslinic acid	104.82	5.83	101.87	0.58	102.18	0.60
2	Corosolic acid	100.06	1.54	103.66	1.47	103.50	0.38
3	Oleanolic acid	106.73	2.78	102.78	0.30	99.87	0.29
4	Ursolic acid	95.47	4.61	106.12	0.36	104.95	1.06
5	β-Amyrin	103.21	3.33	100.52	1.25	100.79	0.17
6	Campesterol	106.32	0.79	102.24	1.95	101.79	0.56
7	α-Amyrin	106.32	1.26	104.25	1.26	100.52	0.40
8	β-Sitosterol	98.71	2.38	101.08	0.87	104.56	0.13
9	Squalene	107.96	0.76	101.60	0.03	103.68	0.97

^1^ Concentrations of all standards were 10 µg/mL. ^2^ Concentrations of level 2 were 250 µg/mL for oleanolic acid, 800 µg/mL for ursolic acid and 50 µg/mL for other triterpene standards. ^3^ Concentrations of level 3 were 500 µg/mL for oleanolic acid, 1600 µg/mL for ursolic acid, and 150 µg/mL for other triterpene standards.

**Table 3:** Determination of precision in cranberry samples.

	Compound	Mean Amount µg/g DW ± SD	Intra-Day Precision (%RSD, *n* = 6)		Inter-Day Precision (%RSD, *n* = 18)		%RSDr	HorRat
			Retention Time	Amount	Retention Time	Amount		
**No.**	***Vaccinium oxycoccos***							
1	Maslinic acid	94.50 ± 2.97	0.10	1.62	0.25	3.14	8.03	0.39
2	Corosolic acid	282.22 ± 5.69	0.11	1.25	0.26	2.05	6.82	0.30
3	Oleanolic acid	1690.11 ± 31.64	0.11	0.78	0.35	1.87	5.21	0.36
4	Ursolic acid	8182.00 ± 121.72	0.10	1.00	0.36	1.49	4.11	0.36
5	β-Amyrin	<LOD	–	–	–	–	–	–
6	Campesterol	66.18 ± 5.88	0.11	3.16	0.44	8.88	8.47	1.05
7	α-Amyrin	162.59 ± 6.41	0.06	1.96	0.53	3.95	7.40	0.53
8	β-Sitosterol	1244.24 ± 19.75	0.07	1.59	0.55	1.71	5.46	0.31
9	Squalene	57.24 ± 2.02	0.08	1.29	0.60	3.53	8.66	0.41
**No.**	***Vaccinium macrocarpon***							
1	Maslinic acid	<LOQ	0.34	–	0.31	–	–	–
2	Corosolic acid	84.91 ± 2.58	0.30	2.07	0.34	2.47	8.16	0.30
3	Oleanolic acid	1118.91 ± 20.82	0.46	1.33	0.43	1.86	5.54	0.34
4	Ursolic acid	5582.68 ± 88.98	0.48	1.21	0.44	1.59	4.36	0.37
5	β-Amyrin	<LOD	–	–	–	–	–	–
6	Campesterol	<LOD	–	–	–	–	–	–
7	α-Amyrin	<LOQ	0.78	–	0.61	–	–	–
8	β-Sitosterol	1176.18 ± 21.34	0.88	0.90	0.68	1.81	5.50	0.33
9	Squalene	10.97 ± 1.23	0.20	8.81	0.56	11.20	11.09	1.01

**Table 4:** Variability of triterpene compounds and phytosterols in samples of different parts of cranberry fruit.

Vaccinium oxycoccos				
Compound	Whole Berry	Peel	Pulp + Seeds	
Maslinic acid	46.5 ± 0.70 ^a^	106.7 ± 1.60 ^a^	<LOD	
Corosolic acid	99.6 ± 1.49 ^a^	281.6 ± 4.22 ^b^	<LOD	
Oleanolic acid	1516.7 ± 22.75 ^c^	2046.2 ± 30.69 ^d^	<LOQ	
Ursolic acid	5222.6 ± 78.34 ^d^	5809.4 ± 87.14 ^e^	115.4 ± 1.73 ^b^	
β-Amyrin	<LOD	<LOQ	<LOD	
α-Amyrin	57.1 ± 0.86 ^a^	106.9 ± 1.60 ^a^	<LOQ	
Campesterol	<LOQ	<LOQ	<LOQ	
β-Sitosterol	1068.3 ± 16.02 ^b^	1208.7 ± 18.13 ^c^	1029.9 ± 15.45 ^c^	
Squalene	48.6 ± 0.73 ^a^	22.9 ± 0.34 ^a^	23.1 ± 0.35 ^a^	
The sum of the compounds identified, µg/g	8059.3 ± 120.89	9582.5 ±143.74	1168.4 ± 17.53	
**Vaccinium macrocarpon**				
**Compound**	**Whole berry**	**Peel**	**Pulp**	**Seeds**
Maslinic acid	64.2 ± 0.96 ^ab^	112.5 ± 1.69 ^a^	<LOD	<LOD
Corosolic acid	167.4 ± 2.51 ^c^	241.4 ± 3.69 ^b^	<LOD	<LOD
Oleanolic acid	1595.1 ± 23.93 ^e^	2562.3 ± 38.43 ^d^	26.2 ± 0.39 ^a^	92.4 ± 1.39 ^a^
Ursolic acid	5735.1 ± 86.03 ^f^	6612.8 ± 99.19 ^e^	176.2 ± 2.64 ^b^	707.7 ± 10.62 ^c^
β-Amyrin	88.6 ± 1.33 ^abc^	110.8 ± 1.66 ^a^	<LOD	336.7 ± 5.05 ^b^
α-Amyrin	130.5 ± 1.96 ^bc^	249.5 ± 3.74 ^b^	<LOD	<LOQ
Campesterol	<LOQ	<LOQ	<LOQ	66.6 ± 1.56 ^a^
β-Sitosterol	1341.5 ± 20.12 ^d^	1220.6 ± 18.31 ^c^	1155.0 ± 17.33 ^c^	2103.5 ± 32.55 ^e^
Squalene	33.7 ± 0.50 ^a^	<LOQ	<LOQ	1116.9 ± 16.75 ^d^
The sum of the compounds identified, µg/g	9155.9 ± 137.34	11,109.9 ± 166.27	1357.5 ± 20.36	4423.7 ± 66.36

Different letters within the same column indicate statistically significant (*p* < 0.05) differences between contents of the identified compounds.

## Data Availability

All data generated during this study are included in this article.

## Sample Availability

Not available.
